# The role of inversion and face masks on simultaneous and delayed face matching tasks

**DOI:** 10.1371/journal.pone.0295407

**Published:** 2024-01-02

**Authors:** Alejandro J. Estudillo, Hoo Keat Wong

**Affiliations:** 1 Bournemouth University, Poole, United Kingdom; 2 University of Nottingham Malaysia, Semenyih, Malaysia; Education University of Hong Kong, HONG KONG

## Abstract

Although it is generally accepted that face recognition relies on holistic processing, it has been suggested that the simultaneous face matching task may depend on a more analytical or featural processing approach. However, empirical evidence supporting this claim is limited. In two experiments, we further explored the role of holistic and featural processing on simultaneous face matching by manipulating holistic processing through inversion and presenting faces with or without face masks. The results from Experiment 1 revealed that both inversion and face masks impaired matching performance. However, while the inversion effect was evident in both full-view and masked faces, the mask effect was only found in upright, but not inverted, faces. These results were replicated in Experiment 2 but, the inversion and mask effects were stronger in delayed face matching than in simultaneous face matching. Our findings suggest that simultaneous face matching relies on holistic processing, but to a smaller extent compared to higher memory-demanding identification tasks.

## Introduction

Faces are the most relevant cues in social situations, so the successful identification of a face is an essential endeavor for our visual system. A long-standing and influential account in face processing research is that face recognition involves holistic processing [1–7]—the integration of facial features in an undecomposed perceptual whole [8–10]. According to some authors, the face inversion effect supports the holistic account of face recognition [3, 11, 12]. This effect shows a disproportional impairment in the identification of inverted faces compared with the identification of any other inverted non-face objects (but see [13]. Following the holistic view of the inversion effect [3, 14, 15], upright faces trigger the automatic integration of the individual facial features into a holistic gestalt. However, inversion breaks this precept causing the face to be processed using an analytical or featural processing approach [3, 14, 15] but see [16]).

Extracting identity information from faces is not only socially relevant, but it is also crucial in security settings, such as photo ID verification. In the classical laboratory version of ID verification—the simultaneous face matching task—observers are presented with two side-by-side faces, and they must indicate whether these face images depict the same or two different identities [17–20]. Although the simultaneous face matching task minimizes the memory requirements for face identification, research has shown that this task is error-prone [21–24]. In fact, error rates of more than 20 percent are frequently reported [25, 26] and the performance of passport control officers with years of experience in ID identification is equally poor compared to that of undergraduate students [22, 27].

In contrast to the long-standing notion that faces are recognized at a global or holistic level, it has been suggested that the simultaneous face matching task is solved using a more analytical or featural processing strategy [28–33]. Some empirical evidence seems to support this argument. For example, prompting observers to match faces using a feature comparison approach improves face matching performance [31, 32]. In fact, some professional ID-verification training is based on this feature-by-feature matching approach [30, 32, 34]. In addition, performance in unfamiliar face matching is positively correlated with tasks involving featural processing, such as inverted face matching [35], figure matching [21] and Navon local processing [36].

Meanwhile, several studies have also shown evidence of holistic processing in the simultaneous face matching. For example, one fixation to the centre of each face of the pair seems to be enough to get a matching accuracy higher than 70% [37]. In addition, although the positive association found between the performance on upright and inverted trials in face matching tasks suggests the use of featural processing [35], in Megreya and Burton’s study [19], an inversion effect was still evident at a group level (see also [30, 32]). Thus, if it is assumed that inversion impairs holistic processing, Megreya and Burton’s findings point to the involvement of holistic processing in simultaneous face matching. However, this argument is based on the comparison between uprights and inverted trials. This comparison between both conditions is problematic as it does not exclude that inversion simply reflects quantitative, rather than qualitative, changes in the processing (see [16]). This problem can be solved by including an additional and converging holistic processing manipulation (see e.g., [14, 38]). In this sense, if two different manipulations engaged similar cognitive processes (i.e., holistic processing), one would expect that they would have little (if any) summative effects on performance. In fact, research has shown that holistic processing reflected by different tasks such as the composite face task (e.g., [6, 38, 39]) and the part-whole task (e.g., [40]) are reduced for inverted faces (see also [14, 41]).

With the increased use of surgical face masks because of the COVID-19 pandemic, several authors have explored how surgical face masks affect face identification [42–46]. For example, Carragher and Hancock [44] adapted the Glasgow Face Matching Test [21] to study the effect of masks on simultaneous face matching (see also [43, 45, 46]). Despite the simultaneous presentation of both faces, face masks also disrupted performance in face matching. Interestingly, using the Cambridge Face Memory—a highly reliable and valid measure of face memory [21, 47–51]—Freud and colleagues [42] showed that the inversion effect is reduced by the presence of face masks. If it is assumed that inverting a face disrupts holistic processing [3, 11, 12] these results suggest that face masks also impair holistic processing.

### The present study

Although previous studies using memory paradigms have shown that face masks reduce the inversion effect [42], it is unknown whether such a finding would also be observed in the face matching task. Given that face masks and inversion seem to impair holistic processing of faces [14, 15, 42], this question is important as it would shed light on the role of holistic processing in the simultaneous face matching task. Specifically, if face matching relies on holistic processing, the inversion, and the presence of masks would impair face matching performance. Importantly, as both manipulations impair holistic processing, they would have little (if any) additional effect on face matching performance. In other words, the inversion and masks effects would be more evident in unmasked and upright faces, respectively.

## Experiment 1

To explore the role of holistic in simultaneous face matching, in Experiment 1 we present full-view and masked pairs of faces in both upright and inverted orientations. We expect to find that both manipulations will impair face matching performance. Importantly, if simultaneous face matching relies on holistic processing, we expect a stronger inversion effect in unmasked faces and a stronger mask effect in upright faces.

### Method

#### Participants

200 students from Bournemouth University participated in this experiment during 2021 Fall term. Participants gave their consent to participate in this study and received course credits as compensation for their time. No identifiable information was collected during this study. Ten participants were removed from further analysis due to performance below chance level and/or abnormally fast response times (< 400 ms), so only 190 participants (159 females) with a mean age of 20 years (SD = 3.39) were included in the data analysis. Retrospective power analysis run with the software MorePower [52] revealed that with 190 participants and a power of .80, we would be able to detect a small two-way interaction (*η^2^*_*p*_ = .04) between orientation and viewing condition. This study was approved by the research ethics committee of Bournemouth University.

#### Stimuli

This experiment used 120 pairs of female and male Caucasian faces from the Glasgow Unfamiliar Face Database [21]. While one of the faces of each pair was a still frame from a video, the other face photograph was taken with a high-quality digital camera (for further details see [20]). All faces were shown in greyscale on a white background. 60 pairs consisted of identity matches (i.e., two pictures from the same identity), while the other 60 pairs were identity mismatches (i.e., two pictures from two different people). For each identity condition, half of the pairs were presented in full-view and, in the other half, both faces were presented with face masks. Face masks were fitted individually for each face pair using Adobe Photoshop. In addition, across the different identity and masking conditions, half of the trials were presented in an upright orientation and the other half consisted of inverted stimuli. Inverted stimuli were created by flipping the images vertically. The allocation of each stimuli pair to the masking and orientation conditions was randomized across participants.

#### Procedure

On each trial, a fixation cross was firstly presented for 1000 ms, followed by two side-by-side face images. Observers were asked to determine whether the two pictures depicted the same or two different people by pressing one of two buttons. After the response, a new trial started. Stimuli presentation was randomized. A demo of this task can be found at https://www.testable.org/experiment/4045/427617/start.

#### Data analysis

Correctly identified match pairs (i.e., hits) and incorrectly identified mismatch pairs (i.e., false alarms) were used to calculate d-prime, a measure of sensitivity [53]. Higher d-prime values reflect a better matching performance. As some participants performed with perfect accuracy, d-prime was corrected using Hautus’ method for extreme values [54]. In addition to conventional frequentist analysis, for the analysis of interactions, we report Bayes Factor (BF) to test the relative support for the alternative and null hypotheses.

### Results

**Fig 1:**
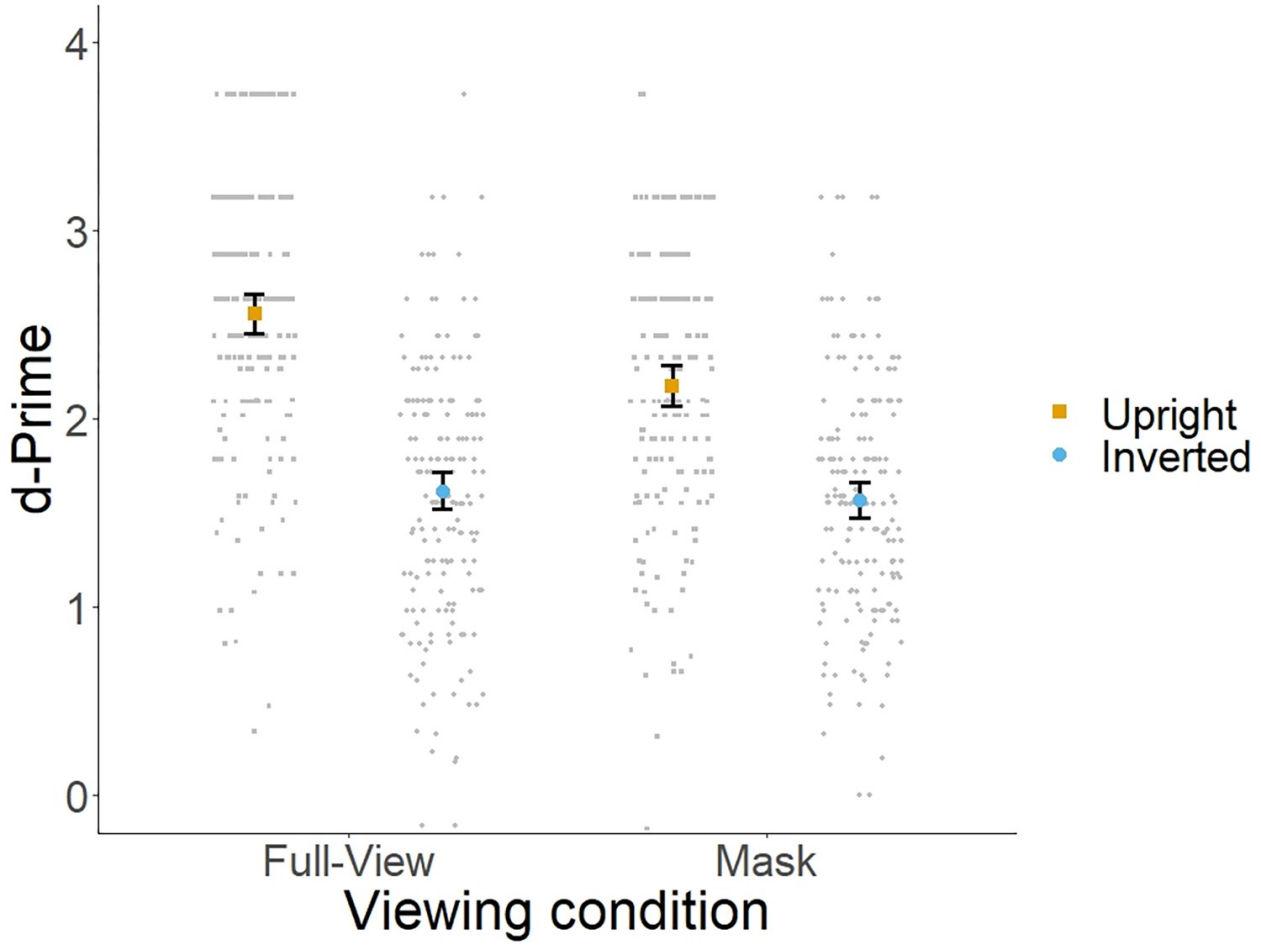
Mean d-prime across viewing and orientation conditions. Error bars represent 95% confidence intervals.

Fig 1 shows the mean d-prime across conditions. A 2 (viewing condition: full-view vs. mask) x 2 (orientation: upright vs. inverted) repeated measures ANOVA was performed. The main effect of viewing condition [*F*(1, 189) = 36.13, *p <* .*001*, η^2^_p_ = .16] and orientation [*F*(1, 189) = 357.30, *p <* .*001*, η^2^_p_ = .65] reached statistical significance. In addition, the interaction between these two factors was also significant [*F*(1, 189) = 19.13, *p <* .*001*, η^2^_p_ = .09]. Fig 1 shows that this interaction arose as a consequence of (1) a stronger inversion effect in the full-view condition compared to the mask condition and (2) a mask effect in upright, but not in inverted trials.

To confirm this pattern, we conducted a simple main effects analysis. For full-view faces, the analysis showed better performance for upright faces (M = 2.56, SD = .74) compared to inverted faces (M = 1.62, SD = .69) [*F*(1, 189) = 299.01, *p <* .*001*, η^2^_p_ = .61]. Bayesian analysis revealed very strong evidence for the differences between upright and inverted faces in the full-view condition (BF_10_ = 3.22e+38). For masked faces, we also observed better performance for upright faces (M = 2.17, SD = .74) compared to inverted faces (M = 1.57, SD = .64) [*F*(1, 189) = 19.13, *p <* .*001*, η^2^_p_ = .09] (BF_10_ = 1.18e+18). However, this effect appears to be relatively smaller compared to the effect observed in full-view faces.

For upright faces, performance was significantly better for full-view (M = 2.56, SD = .74) compared to masked faces (M = 2.17, SD = .74) [*F*(1, 189) = 58.41, *p <* .*001*, η^2^_p_ = .23]. Bayesian analysis provided very strong support for the differences between full-view and masked faces (BF_10_ = 5.78e+9). In contrast, for inverted faces, performance was similar between the full-view (M = 1.61, SD = .69) and mask conditions (M = 1.57, SD = .7644) [F < 1]. In fact, Bayesian analysis revealed substantial evidence for the lack of differences across viewing conditions in inverted trials (BF_01_ = 6.20).

### Discussion

Experiment 1 explored the effect of surgical face masks and inversion on simultaneous face matching. Observers performed a simultaneous face matching task with upright and inverted faces. The faces were also presented with and without face masks. The results can be summarized as follows. First, we found that face masks impaired matching performance in upright trials, but such an effect was not observed in inverted trials. Second, we found clear inversion effects in both the full-view and mask conditions. However, these effects were smaller in the latter, replicating previous findings obtained with face memory paradigms [42].

Although the results of Experiment 1 reflect some differences between inversion and face masks, if it is assumed that both manipulations disrupt the holistic processing of faces, this experiment provides evidence for the involvement of holistic processing in simultaneous face matching tasks. Experiment 1 findings are in agreement with recent reports using face recognition tasks [42], suggesting that both face matching and face recognition require qualitatively similar processes. However, the quantity of holistic processing engaged in a task might be modulated by memory demands. Specifically, while the simultaneous presentation of a pair of faces might promote a more featural approach, the retrieval of a face from memory might require a more unified holistic representation of that face. This hypothesis has been raised by other authors[28, 29, 31, 32]; yet, to the best of our knowledge, it has not been directly tested. Experiment 2 tested this hypothesis by comparing inversion and mask effects under memory and no memory demand conditions.

## Experiment 2

In this experiment, observers performed two different tasks: simultaneous and delayed face matching tasks. In the delayed face matching task, in each trial, two faces are presented sequentially with a short interval of time between them. Thus, to successfully solve the task observers need to retrieve the identity information of the first face from their memory and match this representation to the second face. In contrast, in the simultaneous face matching task, as both faces are presented together side-by-side, memory demands are virtually abolished. If the magnitude of holistic processing is modulated by memory demands, we would expect stronger mask and inversion effects in the delayed face matching compared to the simultaneous face matching task.

### Participants

132 participants from Bournemouth University took part in this experiment during the winter and spring terms in 2022. They received course credits for their time and provided their consent to participate. No identifiable information was collected during this study. Two participants were removed from further analysis due to performance below chance level and/or abnormally fast response times (< 400 ms), so our final sample comprised 130 participants (106 females) with a mean age of 21 years (SD = 5.47). Retrospective power analysis run with the software MorePower [52] revealed that with 130 participants and a power of .80, we would be able to detect a small two-way interaction (η^2^_p_ = .05) between task and orientation or viewing condition.

### Stimuli

The same 120 pairs of female and male Caucasian faces from the Glasgow Unfamiliar Face Database used in Experiment 1 were used in Experiment 2. Half of the stimuli (60) were utilized for the simultaneous face matching task, while the other half were assigned to the delayed face matching task. The allocation of each face pair to each task was counterbalanced among participants. Across these two tasks, half of the stimuli (30) were identity match trials, and the remaining half were identity mismatch trials. Additionally, within the two tasks and identity conditions, half of the trials (15) were presented in full view, while the other half were presented with a face mask. This resulted in a total of 15 trials per condition for both upright and inverted trials. To address this imbalance, half of the participants were presented with 7 upright trials and 8 inverted trials per condition, while the other half of the participants were presented with 8 upright and 7 inverted trials per condition.

### Procedure

This experiment consisted of two tasks: a simultaneous face matching task and a delayed face matching task. The order of the tasks was randomized across participants and a self-paced break was introduced after the first task. Each task had a total of 60 trials. The simultaneous face matching task was identical to that of Experiment 1. The events of the delayed face matching task were as follows. A fixation cross was first presented for 1000 ms, followed by one central face for 1500 ms. After a four-seconds blank screen, a second face appeared, and participants were asked to decide whether the two faces depicted the same or two different people. After the response, a new trial started. A demo of this experiment can be found at https://www.testable.org/experiment/4045/665944/start

### Results

**Fig 2:**
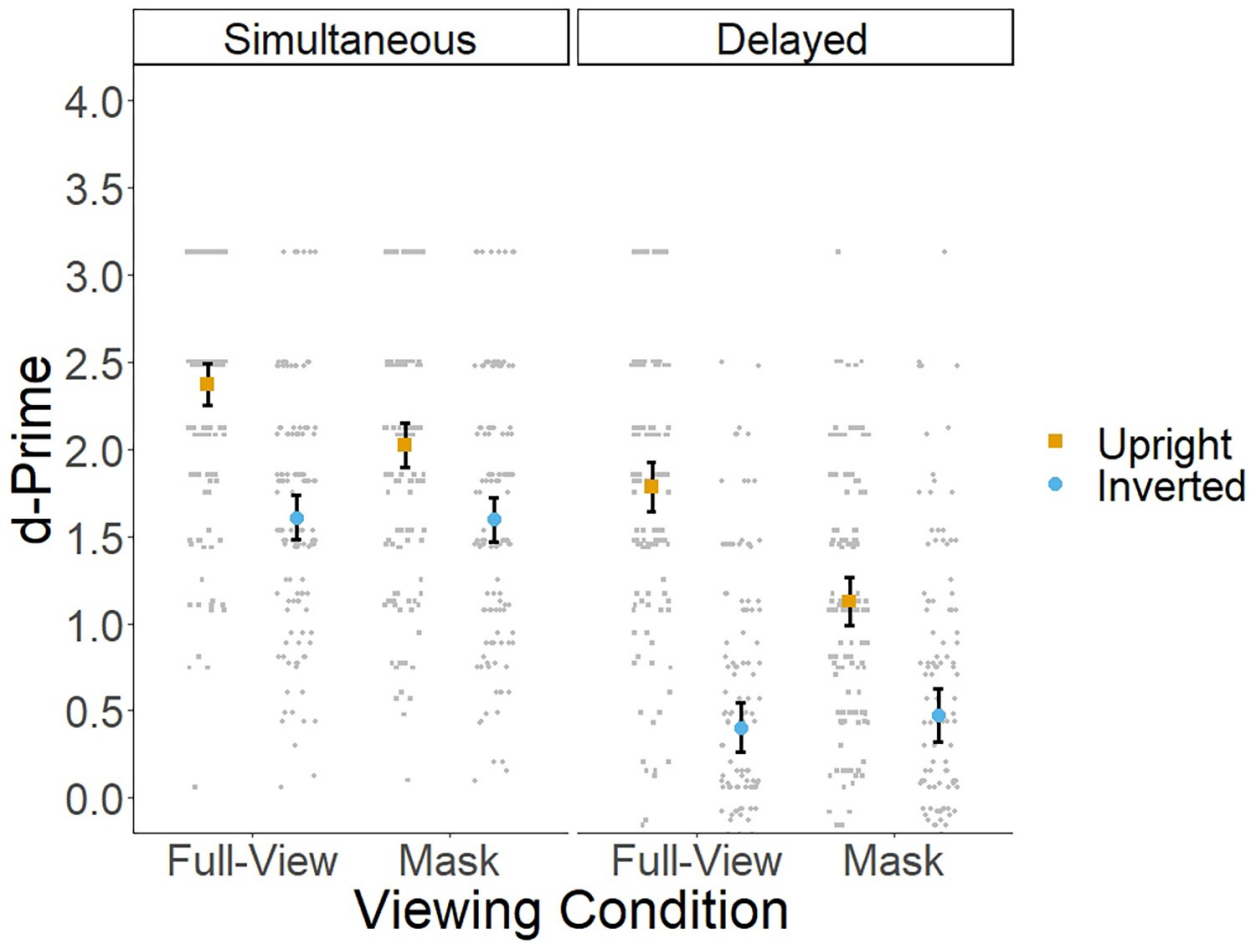
Mean d-prime across tasks, viewing and orientation conditions. Error bars represent 95% confidence intervals.

The same analysis protocol as in Experiment 1 was used in Experiment 2. Fig 2 and Table 1 show the mean d-prime across conditions and tasks. A 2 (task: simultaneous vs. delayed) x 2 (viewing condition: full-view vs. mask) x 2 (orientation: upright vs. inverted) repeated measures ANOVA revealed main effects of task [*F*(1, 129) = 440.15, *p <* .*001*, η^2^_p_ = .77], viewing [*F*(1, 129) = 36.76, *p <* .*001*, η^2^_p_ = .22] and orientation [*F*(1, 129) = 258.01, *p <* .*001*, η^2^_p_ = .66]. Although the two-way interaction between task and viewing condition did not reach statistical significance [*F*(1, 129) = 3.11, *p =* .*08*], the two-way interaction between viewing condition and orientation [*F*(1, 129) = 50.34, *p <* .*001*, η^2^_p_ = .28] and the three-way interaction between task, viewing condition and orientation [*F*(1, 129) = 6.81, *p <* .*01*, η^2^_p_ = .05] reached statistical significance.

**Table 1:** Mean d-prime across tasks, viewing and orientation conditions.

Viewing Condition	Task	Orientation	Mean	SD
Full-View	Simultaneous	Upright	2.37	0.68
		Inverted	1.60	0.72
	Delay	Upright	1.78	0.81
		Inverted	0.40	0.81
Mask	Simultaneous	Upright	2.02	0.72
		Inverted	1.59	0.72
	Delay	Upright	1.12	0.78
		Inverted	0.47	0.87

Fig 2 suggest that the three-way interaction arose as a consequence of stronger inversion and mask effects in the delayed face matching task compared to the simultaneous face matching task. To further explore this interaction, we conducted separate ANOVAs for each viewing and orientation conditions. For full-view faces, the main effect of task [*F*(1, 129) = 233.37, *p <* .*001*, η^2^_p_ = .64], orientation [*F*(1, 129) = 307.08, *p <* .*001*, η^2^_p_ = .70], and the interaction between these two factors [*F*(1, 129) = 26.89, *p <* .*001*, η^2^_p_ = .17] reached statistical significance. Performance was significantly higher for upright compared to inverted faces (see Table 1) in both simultaneous [*F*(1, 129) = 105.01, *p <* .*001*, η^2^_p_ = .45, BF_10_ = 3.73e+16] and delayed face matching tasks [*F*(1, 129) = 211.20, *p <* .*001*, η^2^_p_ = .62, BF_10_ = 2.03e+32]. However, this effect appears to be larger in the delayed compared to the simultaneous face matching task. Performance was also higher for the simultaneous compared to the delayed face matching tasks in both upright [*F*(1, 129) = 52.03, *p <* .*001*, η^2^_p_ = .28, BF_10_ = 8.69e+8] and inverted conditions [*F*(1, 129) = 199.01, *p <* .*001*, η^2^_p_ = .60, BF_10_ = 2.14e+29], but the differences across tasks are more pronounced in the inverted condition. For masked faces, performance was higher in the simultaneous compared to the delayed face matching task [*F*(1, 129) = 199.01, *p <* .*001*, η^2^_p_ = .60] and in the upright compared to the inverted condition [*F*(1, 129) = 71.40, *p <* .*001*, η^2^_p_ = .35]. However, the interaction between task and orientation for masked faces did not reach statistical significance [*F*(1, 129) = 3.48, *p =* .*06*].

For uprights faces, the main effect of task [*F*(1, 129) = 146.74, *p <* .*001*, η^2^_p_ = .53], viewing condition [*F*(1, 129) = 91.42, *p <* .*001*, η^2^_p_ = .41], and the interaction between these two factors [*F*(1, 129) = 9.69, *p <* .*001*, η^2^_p_ = .07] reached statistical significance. Performance was significantly higher for full-view compared to masked faces in both simultaneous [*F*(1, 129) = 28.19, *p <* .*001*, η^2^_p_ = .18, BF_10_ = 27557] and delayed face matching tasks [*F*(1, 129) = 70.29, *p <* .*001*, η^2^_p_ = .35, BF_10_ = 1.30e+11]. However, the effect of the mask appears to be larger in the delayed compared to the simultaneous face matching task. Performance was also higher in the simultaneous compared to the delayed face matching tasks in both full-view [*F*(1, 129) = 52.03, *p <* .*001*, η^2^_p_ = .28, BF_10_ = 7.13e+8] and masked conditions [*F*(1, 129) = 140.10, *p <* .*001*, η^2^_p_ = .52, BF_10_ = 2.01e+20], but the differences across tasks are more pronounced in the mask condition. For inverted faces, performance was higher in the simultaneous compared to the delayed face matching task [*F*(1, 129) = 275.07, *p <* .*001*, η^2^_p_ = .68]. However, neither the main effect of viewing condition nor the interaction between viewing condition and task reached statistical significance [both *Fs* < 1].

### Discussion

Experiment 2 explored whether memory demands modulate the holistic processing of faces. Observers performed both a simultaneous and a delayed face matching with upright and inverted trials. In addition, faces were presented with and without face masks. Although inversion and mask effects were present in both the delayed and the simultaneous face matching tasks, these effects were stronger in the former compared to the latter. Altogether these findings suggest that memory demands modulate the amount of holistic processing. Specifically, holistic processing seems to be more relevant under higher memory-demanding identification tasks.

## General discussion

In two different studies, we explored the role of holistic processing in simultaneous face matching tasks by combining two converging manipulations of holistic processing. In Experiment 1, observers matched full-view and masked faces presented in both upright and inverted orientations. Although the inversion effect was evident in both the full-view and the masked conditions, this effect was clearly stronger for full-view faces. Experiment 1 also showed a mask effect, but only in upright faces. This pattern of results was replicated in Experiment 2 but, interestingly, the mask and the inversion effects were larger in the delayed matching task than in the simultaneous matching task. Altogether our results suggest that simultaneous face matching relies on holistic processing, but to a lesser extent compared to delayed face matching.

While it has been commonly assumed that face recognition relies on holistic processing [8, 10], some evidence has questioned the role of holistic processing in simultaneous face matching and suggests that this task relies on a more featural processing approach [29–33]. However, these studies either did not directly manipulate holistic processing per se or their conclusions are based on simple correlations between two conditions (e.g., upright and inverted trials), which might reflect processes other than holistic and featural processing (but see [30, 32]). In contrast, using two different holistic processing manipulations, the results of our two experiments show a clear involvement of holistic processing in simultaneous face matching.

Interestingly, both inversion and mask effects seem to be modulated by memory demands. Specifically, the effects of inversion and masking were more pronounced in the delayed face matching task compared to the simultaneous face matching task. Importantly, the decrease in performance observed cannot be solely attributed to the (close to) ceiling effects in the full-view upright simultaneous face matching. In fact, due to this high performance in the full-view upright simultaneous face matching condition, any inversion or masking effect would potentially have a greater impact on performance in the simultaneous task compared to the delayed face matching task. However, as our results show, these effects are larger in the delayed face matching task. Therefore, considering that the only difference between both was the increased memory demands in the delayed face matching task, the results of Experiment 2 suggest that higher memory demands increase the reliance on holistic processing.

One might wonder about the reasons behind these differences between tasks. We believe that these differences can be explained by the pictorial nature of the simultaneous face matching task [35]. One critical difference between face identification tasks with memory demands and the simultaneous face matching tasks is the actual access to the identity of the faces. In fact, previous research has shown that access to identity only occurs in face memory paradigms but not in the simultaneous face matching [55]. Thus, identifying a face under memory demands conditions would require an abstract structural representation of the face [56, 57]. It is possible that in this abstract representation the configuration of facial features is more important than the features themselves [58]. On the contrary, when both faces are presented simultaneously, this abstract representation is not needed, so observers would require less holistic processing to solve the task.

It is important to note that our results do not question the benefits of featural processing training on the simultaneous face matching task [31, 32, 59]. Instead, our results suggest that holistic processing is a mandatory process in simultaneous face matching. Nevertheless, an intentional featural processing strategy can also be used to aid the identity decision. This featural route, which has been previously proposed by different authors [57, 60], acts as a secondary route that operates in a controlled way and that can be trained. When faces are presented simultaneously, observers can freely compare specific features between both faces. However, this is not the case in other face identification tasks that demand higher memory resources. In such tasks, observers need to compare the displayed face with the memory representation of a previously learned face. Therefore, it seems reasonable to assume that this featural route would have a stronger impact on simultaneous face matching tasks compared to higher memory-demanding identification tasks. However, this remains an open question for further research.

It must be noted that inversion does not directly manipulate holistic processing, but the drop in identification in inverted faces is taken as evidence of holistic processing of upright faces [15, 61]. A significant body of previous research has supported this assumption that inversion does impair holistic processing of faces [3, 11, 12], therefore reduced inversion effects for masked faces seem to suggest that face masks also disrupt holistic processing of faces [42]. However, it could also be possible that inversion and face masks disrupt other common cognitive processes and the absence of mask effects in inverted trials might reflect the disruption of these other processes. To formally investigate this issue, future research could incorporate online manipulations of holistic and featural processing, such as gaze-contingent [14] or dynamic aperture [62] paradigms, in addition to masked and inverted faces.

Importantly, our results reveal an important difference between inversion and face masks. Specifically, while inverting a face impaired performance on both full-view and masked faces, face masks only disrupted the performance on upright faces. This pattern of results probably reflects that, although both inversion and face masks disrupt the holistic processing of faces, the degree of this disruption is higher in inverted compared to masked faces. In fact, research has shown that inversion impairs the discrimination of the relational information between the different facial features [62, 63]. Conversely, as face masks only cover the bottom part of the face, some of this relational information is still available in masked faces (e.g., the distance between both eyes) [3, 64–66].

One important shortcoming of the current study must be noted. In both experiments, the number of female participants was considerably higher. Previous research has shown a female advantage in face identification tasks [67, 68], so it could be argued that part of our results can be explained by this sex imbalance. Given this large imbalance between males and females in our experiments, we lack enough statistical power to formally address this issue. However, it is important to note that in face matching tasks the female advantage appears to be consistent across different conditions, including matching full-view faces and more challenging conditions involving external or internal features only [63]. This consistency suggests that the effects of masks and inversion should also be consistent, thereby maintaining the overall effects. In fact, we recently reported a female advantage when identifying masked faces [64].

In conclusion, both face inversion and the presence of face masks impaired participants’ ability to perform simultaneous face matching. However, these effects were smaller compared with the delayed face matching task. Altogether our study suggests that while holistic processing is still a significant factor in simultaneous face matching, its influence might be somewhat diminished compared to more memory-demanding tasks like the delayed face matching task. Future research investigating the mechanisms behind the interaction between holistic processing and memory demands in different face identification tasks could inform face training programs or contribute to our understanding of cognitive processes in face perception.
